# Merging Digital Medicine and Economics: Two Moving Averages Unlock Biosignals for Better Health

**DOI:** 10.3390/diseases6010006

**Published:** 2018-01-06

**Authors:** Mohamed Elgendi

**Affiliations:** 1 Department of Obstetrics & Gynecology, University of British Columbia and BC Children’s & Women’s Hospital, Vancouver, BC V6H 3N1, Canada; 2 School of Electrical and Computer Engineering, University of British Columbia, Vancouver, BC V6T 1Z4, Canada; mohamed.elgendi@cw.bc.ca; Tel.: +1-604-600-4139

**Keywords:** data analysis, global health, digital health, mobile health, simplicity, algorithms

## Abstract

Algorithm development in digital medicine necessitates ongoing knowledge and skills updating to match the current demands and constant progression in the field. In today’s chaotic world there is an increasing trend to seek out simple solutions for complex problems that can increase efficiency, reduce resource consumption, and improve scalability. This desire has spilled over into the world of science and research where many disciplines have taken to investigating and applying more simplistic approaches. Interestingly, through a review of current literature and research efforts, it seems that the learning and teaching principles in digital medicine continue to push towards the development of sophisticated algorithms with a limited scope and has not fully embraced or encouraged a shift towards more simple solutions that yield equal or better results. This short note aims to demonstrate that within the world of digital medicine and engineering, simpler algorithms can offer effective and efficient solutions, where traditionally more complex algorithms have been used. Moreover, the note demonstrates that bridging different research disciplines is very beneficial and yields valuable insights and results.

## 1. Introduction

Few scientists have the opportunity to revisit and rethink the complexity component of algorithm development. With the competing demands of research, scientists also have limited bandwidth for investigating the integration of concepts from other disciplines that could yield simple algorithm solutions. Recently, an interesting paper [1] published at the University of British Columbia explored this concept and demonstrated how the idea of taking a simple method regularly used in economics can be applied to algorithm development in digital medicine.

## 2. Simplicity: A Moving Average

The concept of a moving average is quite intuitive and relatively easy to understand and implement. Essentially, a moving average acts as a filter and eliminates unnecessary data. Upon further investigating this seemingly simple concept, one can see that it has more depth than initially thought. Using this concept and with the intent to demonstrate the power of simplicity and concept integration, a multidisciplinary research team recently published impactful results on climate change [2].

## 3. Economic Concept: Two Moving Averages

In economics, one moving average is commonly used by traders to identify trends and directions for whatever the trader is looking at. To determine the selling and buying points, economists and traders use crossover signs for decision making.

**Figure 1:**
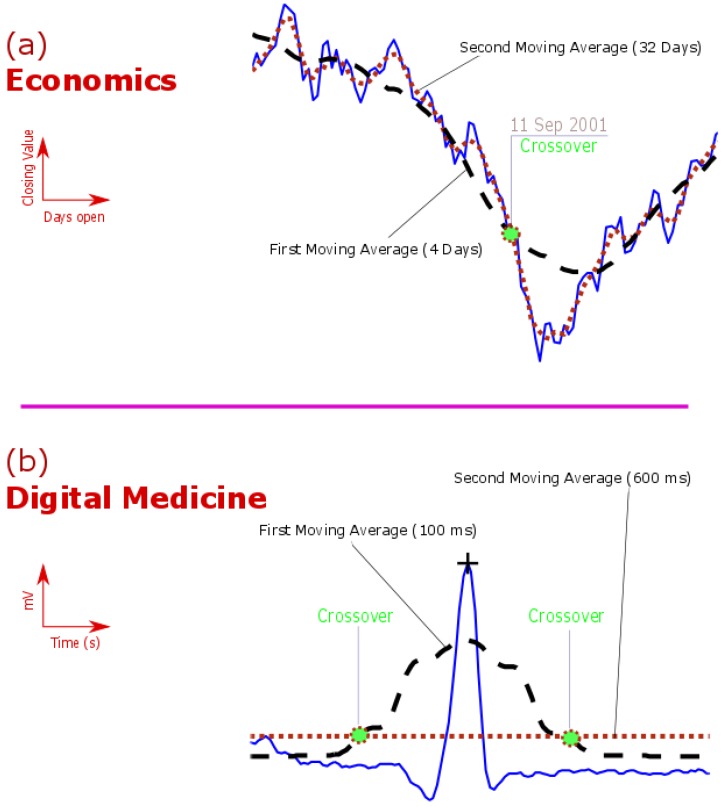
Demonstration of the use of Two Moving Averages in Economics and Digital Medicine. (**a**) Detection of the 11 September 2001 collapse in the NASDAQ composite index for the calendar year 2001 (**b**) Detection of QRS complex in the electrocardiogram signal.

To generate crossover signs, the two moving averages need to have two different window sizes. One moving average has to be faster (shorter) than the other, as shown in Figure 1a. Looking at the NSADAQ composite index for the calendar year 2001 with the use of two moving averages 4 days and 32 days, we can see the collapsing point on 11 September 2001.

## 4. Analogy Application between Two Disciplines

Biomedical signals are time series data (data collected over time) containing features that represent periodical physiological events (events repeated approximately every second). For example, each event of the heart cycle is reflected by a biosignal, or a wave. Typically, biomedical engineers try to detect these waves automatically in order to make the diagnosis process more automated for clinicians. Detection of these waves through the use of an algorithm is a difficult task and traditionally has been accomplished with the development of complicated algorithms.

Transferring knowledge between different disciplines moves at a relatively slow pace and is often approached with hesitation for a variety of reasons, such as discomfort in understanding concepts from another discipline. Approaching fluctuation analysis in economics, for example, may not be attractive to digital medicine scientists who typically work with medical data This fear or discomfort is somewhat displaced, however, as there are areas of similarity in many fields such as can be seen in economical and biomedical data; both use periodical data that contain repeated trends (cycles). This overlap is also seen with the two moving averages concept explained previously, and thus combining the concept with time series biomedical data seems logical and practical. In the fields of biochemistry and biophysics, and astronomy we can see the use of two moving averages as well (one moving average shorter in duration than the other) with an eye patch analysis [3]. However, biomedical signals are far richer, and the application of the two moving averages could yield even more impactful results. The implementation of two moving averages requires minimal computational resources in contrast to most of the currently used machine-learning approaches.

As shown in Figure 1b, the two moving averages approach was able to demarcate the QRS complex (refers to one heartbeat) via two crossovers. In other words, the area in between the two crossovers is the area that contains the QRS complex and therefore searching for the maximum amplitude to ensure beat detection can be easily achieved. In fact, the application of the two moving averages in digital medicine applications achieved higher accuracy than more sophisticated algorithms, especially in detecting:

(1) QRS complexes in ECG signals: The two moving averages obtained a sensitivity (SE) of 99.29% and a positive predictivity (+P) of 98.11% over the first lead of the validation databases (10 databases with a total of 1,179,812 beats). When applied to the well-known MIT-BIH Arrhythmia Database, an SE of 99.78% and a +P of 99.87% were scored [4] and the improved version accomplished a SE of 99.90% and +P of 99.84% [5]. This simple approach outperformed most of the well-known QRS detectors, such as Pan–Tompkins [6] (SE of 90.95% and +P of 99.56%) and Hamilton–Tompkins [7] (SE of 99.69% and +P of 99.77%), which are more complex algorithms in terms of implementation and processing time.
(2) T waves in ECG signals: Over the MIT-BIH Arrhythmia Database, the two moving averages were able to achieve a SE of 99.86% and a +P of 99.65% [8]. Unfortunately we cannot compare the performance with any other methods on the same dataset as the annotation of T-waves was published in 2015. However, the results are very promising, as the overall accuracy on arrhythmic ECG signals is more than 99.7% [9].
(3) Systolic waves in PPG signals: The two moving averages were able to detect systolic waves in 40 subjects measured at rest and after three heat stress simulations containing 5071 heartbeats, with an overall SE of 99.89% and +P of 99.84% [10]. This simple approach slightly outperformed existing algorithms, such as Billauer’s [10] (SE of 99.88% and +P of 98.69%), Li’s [10] (SE of 97.9% and +P of 99.93%) and Zong’s [10] (SE of 99.69% and +P of 99.71%).
(4) *a* and *b* waves in APG signals: The two moving averages demonstrated an overall SE of 99.78% and a +P of 100% for detecting *a* waves and overall SE of 99.78% and +P of 99.95% for detecting *b* waves [11]. There are no *a* and *b* waves detectors to compare the algorithm with, as it is a new area of investigation in the field of PPG signal analysis. However, the results are very promising and the accuracy is more than 98%.
(5) *c*, *d* and *e* wave detection in APG signals: The performance of the two moving averages was tested on 27 PPG records collected during rest and after 2 h of exercise, resulting in 97.39% SE and 99.82% +P [12]. The proposed algorithm was not compared to other algorithms, as it is a new area of investigation in the field of PPG signal analysis. However, the results are very promising, as the overall accuracy achieved is more than 97%.
(6) First and second heart sounds: The SE and +P of the two moving averages detectors were 70% and 68%, respectively, for heart sounds collected from children with pulmonary artery hypertension [13]. Again, the proposed algorithm outperformed existing algorithms, such as Liang’s [13] (SE of 59% and +P of 42%), Kumar’s [13] (SE of 19% and +P of 12%), Wang’s [13] (SE of 50% and +P of 45%) and Zhong [13] (SE of 43% and +P of 53%).

## 5. Conclusions

Based on an environmental scan of research, there is a lack of investigation into simple approaches for larger applicability across different disciplines. Given the significant benefits of developing simple approaches (e.g., lower resource consumption), it is essential for researchers to try and implement simplistic and scalable approaches into their time series analysis, such as can be seen with two moving averages. Simple but effective methods are crucial to widespread sharing and success in science. The economical-based method gives more insight into data and offers a fast solution to big data analysis. Researchers who are (or will) apply this approach can benefit in many ways, and it may open new perspectives with data analysis leading to more accurate conclusions.
